# Nutritional consequences of breeding away from riparian habitats in Bank Swallows: new evidence from multiple endogenous markers

**DOI:** 10.1093/conphys/coaa140

**Published:** 2021-01-19

**Authors:** Corrine S V Génier, Christopher G Guglielmo, Greg W Mitchell, Myles Falconer, Keith A Hobson

**Affiliations:** 1 Department of Biology, University of Western Ontario, 1151 Richmond Street, London, Ontario N6A 3K7, Canada; 2 Environment and Climate Change Canada, Wildlife Research Division, 1125 Colonel By Drive, Ottawa, Ontario K1A 0H3, Canada; 3 Birds Canada, 115 Front Rd., P.O. Box 160, Port Rowan, Ontario N0E 1M0, Canada; 4 Environment and Climate Change Canada, Wildlife Research Division, 11 Innovation Blvd., Saskatoon, Saskatchewan S7N 3H5, Canada

**Keywords:** Bank Swallows, diet quality, DNA metabarcoding, fatty acids, population decline, stable isotopes

## Abstract

The Bank Swallow (*Riparia riparia*), a threatened species in Canada, breeds primarily in banks at lakeshores and rivers and in artificial (typically inland) aggregate mining pits. Inland pits may be ecological traps for this species, but relative dietary trade-offs between these two nesting habitats have not been investigated. The availability of aquatic emergent insects at lakeshores may have associated nutritional benefits for growing nestlings due to increased omega-3 fatty acids (FAs) in prey. We compared the diets of juvenile swallows from lakeshore and inland pit sites using assays of stable isotope values (δ^13^C, δ^15^N, δ^2^H) of feathers, faecal DNA metabarcoding and blood plasma FAs. Colony proximity to Lake Erie influenced the use of aquatic versus terrestrial insects by Bank Swallow adults and juveniles. Feather δ^2^H was particularly useful as a tracer of aquatic emergent versus terrestrial prey, and inland juveniles had feathers enriched in ^2^H, reflective of diets composed of fewer aquatic emergent insects. DNA metabarcoding of juvenile and adult faecal material indicated that lakeshore birds consumed more aquatic-emergent chironomids than inland birds. Lakeshore juveniles had elevated plasma omega-3 eicosapentaenoic acid levels compared with inland pit-breeding birds. We discuss the need to consider ‘nutritional landscapes’ and the importance of this concept in conservation of declining species and populations.

## Teaser

Bank Swallows (*Riparia riparia*) breed naturally in riparian habitats but also at inland human-made aggregate pits. Based on three endogenous markers used to track diet, we demonstrate that inland nesting results in nutritional consequences for young birds due to reduced provisioning of omega-3 fatty acids.

## Introduction

Aerial insectivorous birds have declined more precipitously than any other passerine group in North America (Smith *et al.*, 2015; Sauer *et al.*, 2017; North American Bird Conservation Initiative Canada, 2019; Rosenberg *et al.*, 2019). Within this guild, the Bank Swallow (*Riparia riparia*) has shown the second-greatest decline of all species (−4.9% per year; Sauer *et al.*, 2017) and has been designated as threatened in Canada (COSEWIC, 2013; North American Bird Conservation Initiative Canada, 2019). Although causes for declines in aerial insectivores are diverse (Nebel *et al.*, 2010; Michel *et al.*, 2016), much attention has been paid to potential global declines in aerial insects (Møller, 2019; Sánchez-Bayo and Wyckhuys, 2019) and diet quality as defined by concentrations of polyunsaturated fatty acids (PUFAs) (Twining *et al.*, 2016a). As their common and scientific names suggest, Bank Swallows historically bred almost exclusively at banks in riparian habitat where they undoubtedly fed to a large extent on aquatic emergent insects with high omega-3 fatty acid (FA) content (Garrison, 1999; Twining *et al.*, 2018a). More recently, Bank Swallows have established colonies at man-made inland aggregate mining pits (Hjertaas, 1984; Garrison, 1999; Falconer *et al.*, 2016) far from riparian zones. If diets fed to nestlings at inland pits are of poorer quality due to lower quantities of omega-3 FAs in terrestrial insects than aquatic emergent insects (Hixson *et al.*, 2015; Twining *et al.*, 2018a), then such inland pits could be ‘nutritional traps’ and so be contributing to population declines.

Tracing diets in wild birds is immensely challenging. However, the use of biogeochemical markers such as naturally occurring stable isotope ratios in avian tissues can assess diet composition and resource partitioning and trace animal origins (Hobson, 1999a; Kelly, 2000; Ronconi *et al.*, 2014). In addition, assays of essential and non-essential FAs of consumer tissues have provided new means of inferring diet composition, diet quality and resource allocation (Iverson, 2009; Klaiman *et al.*, 2009; Wang *et al.*, 2010; Twining *et al.*, 2016a). The advent of faecal DNA metabarcoding also now provides unprecedented taxonomic information on short-term diets of individual birds (Zeale *et al.*, 2010; Pompanon *et al.*, 2012). These endogenous, time-integrated techniques allow important insight into diets without the need for excessive handling or disturbance at colonies. Such approaches are well suited for studying contrasting avian diets where source insects may be derived from either aquatic or terrestrial environments as is the case for Bank Swallows breeding in riparian versus inland sites.

Carbon stable isotope ratios (depicted as δ^13^C) typically reflect the isotopic composition of primary producers in food webs and clearly define C3, C4 and CAM photosynthetic pathways (O’Leary, 1988; Fogel and Cifuentes, 1993; Adiredjo *et al.*, 2014). However, δ^13^C values may also differ between freshwater- and terrestrial-based food webs, as primary producers in freshwater habitats can often be depleted in ^13^C compared with those supporting terrestrial systems (France, 1995; Doucett *et al.*, 1996). However, the use of this isotope to consistently delineate terrestrial versus aquatic food webs has been debated (France, 1995; Doucett *et al.*, 1996; Clementz and Koch, 2001; Voigt *et al.*, 2015). Nonetheless, δ^13^C measurements have successfully been used to indicate aquatic diet sources for spiders and herptile predators in riparian zones (Walters *et al.*, 2008).

Stable hydrogen isotope ratios are potentially of greater utility in differentiating between aquatic and terrestrial sources of nutrients to avian diets (δ^2^H; Vander Zanden *et al.*, 2016). Local evaporative conditions in terrestrial plants result in enrichment of ^2^H in terrestrial plants compared with aquatic plants that are embedded in water with no evapotranspiration (Wershaw *et al.*, 1966; Fogel and Cifuentes, 1993; Doucett *et al.*, 2007). In addition, aquatic algae discriminate against the heavier isotope more so than terrestrial plants (Doucett *et al.*, 2007). Unless aquatic systems are prone to significant evaporation, aquatic freshwater food webs are expected to be depleted in ^2^H compared with adjacent terrestrial food webs. In Germany, insectivorous bats differentially feeding on terrestrial and aquatic emergent insects were isotopically well separated by lower δ^2^H values in aquatic diets than terrestrial diets (Voigt *et al.*, 2015).

Stable nitrogen isotope (δ^15^N) values can be informative in differentiating between aquatic and terrestrial food webs (Jardine *et al.*, 2012) but are also influenced by a variety of biological (Fogel and Cifuentes, 1993; Casciotti, 2009) and anthropogenic processes in agro-ecosystems (Szpak, 2014), making it difficult to generalize their use as a reliable tracer. Agricultural practices involving the use of fertilizer can cause enrichment in ^15^N, whereby manure-based fertilizer tends to be higher in ^15^N than synthetic fertilizer (Bateman *et al.*, 2005; Szpak, 2014). Once the fertilizer is applied, ammonia volatilization may cause lighter nitrogenous compounds to be lost from soils (Ma *et al.*, 2010) resulting in soils enriched in ^15^N (Hobson, 1999b; Pardo and Nadelhoffer, 2010). The excess deposition and draining of nitrogenous fertilizers from agricultural fields may alter the isotopic composition of regional food webs by elevating tissue δ^15^N of consumers from waterbodies subject to agricultural run-off (Harrington *et al.*, 1998; Hebert and Wassenaar, 2001).

Faecal DNA metabarcoding is a non-invasive method of assessing dietary prey items at the species level for an individual predator (Zeale *et al.*, 2010). For example, Zeale *et al.* (2010) used faecal DNA metabarcoding to accurately identify 37 prey items of insectivorous bats to the genus level. Other studies have successfully used faecal DNA metabarcoding to examine diets of western bluebirds (*Sialia mexicana*; Jedlicka *et al.*, 2013), rufous hummingbird (*Selasphorus rufus*; Moran *et al.*, 2019) and barn swallows (*Hirundo rustica*; McClenaghan *et al.*, 2019). While providing important taxonomic information, the technique is typically non-quantitative.

FAs, carboxylic acid with a saturated or unsaturated chain of 4–24 carbon atoms, are the building blocks of lipids. PUFAs are longer-chain unsaturated FAs including omega-3, -6 and -9 FAs. Unlike terrestrial plants, aquatic algae are able to synthesize large amounts of omega-3 eicosapentaenoic acid (20:5n3 EPA) and docosahexaenoic acid (22:6n3 DHA) from alpha-linolenic acid (18:3n3 ALA) (Hixson *et al.*, 2015). Omega-3 FAs then accumulate in aquatic organisms from algae to invertebrates to higher-order predators (Hixson *et al.*, 2015). As a result, aquatic emergent insects are rich in omega-3 FAs such as EPA and DHA compared with terrestrial insects (Hixson *et al.*, 2015; Twining *et al.*, 2018a). Although omega-3 and -6 precursors of ALA and linoleic acid (18:2n6 LA) are abundant in terrestrial insects (Hixson *et al.*, 2015), nestling tree swallows are inefficient at converting ALA to the longer-chain FAs, EPA and DHA (Twining *et al.*, 2018b). Thus, tree swallows accumulate EPA and DHA from aquatic emergent insects, thereby increasing nestling health and fledging success compared with nestlings provisioned by more terrestrial insects with fewer omega-3 FAs (Twining *et al.*, 2016b, 2018a). Diet quality also had greater benefits than food quantity for tree swallow nestling growth and health (Twining *et al.*, 2016b; see also Gerrard and St. Louis, 2001). Tree swallow nestlings fed high omega-3 diets, regardless of food quantity provided, were in better body condition, grew more quickly, had increased immunocompetence and had decreased metabolic rates compared with nestlings fed low-quality diets (Twining *et al.*, 2016b).

Our study aimed to evaluate the diet composition of Bank Swallows nesting at natural lakeshore sites versus inland mining aggregate pits. We hypothesized that lakeshore- and inland-nesting birds would have different diets, with inland pit individuals having lower levels of aquatic emergent prey in diets compared with those at lakeshores. Diet composition was determined by three independent measures. Values of δ^13^C, δ^15^N and δ^2^H were expected to separate diets based on aquatic and terrestrial sources. FAs were used to assess diet quality and, again, we expected this to reveal higher inputs of aquatic insects at lakeshore sites. DNA metabarcoding was carried out to provide detailed taxonomic information on diet to confirm consumption of aquatic emergent insects.

## Materials and methods

### Field sampling

**Figure 1:**
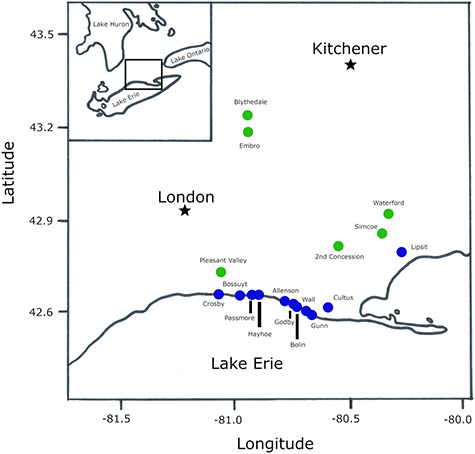
Locations of Bank Swallow sites in the Lake Erie region used in our study 2017–18.

All animal work was approved by the University of Western Ontario’s Animal Care Committee (AUP 2017-005) and by the Canadian Wildlife Service in accordance with applicable regulations. Bank Swallows were sampled from southwestern Ontario, Canada, at colonies along the banks of Lake Erie and at aggregate pits within 70 km of the lake in 2017 and 2018 (Fig. 1). Sites visited differed between years depending on landowner’s consent (Supplementary Table S1). Adult Bank Swallows were captured during their incubation period from late May to mid-June. Recently fledged hatch-year birds (hereafter juveniles) were sampled from late June to mid-July. Free-flying adults and recently fledged juveniles were captured using a framed mist net dropped from above the bank and in front of the burrows. Birds were banded with a Canadian Wildlife Service numbered aluminum leg band and standard measurement was taken.

Faecal matter was opportunistically collected when birds were handled for banding then transferred into scintillation vials with 95% ethanol and stored in a field cooler. Faecal samples were transferred within 24 hours into a −40^o^C freezer. Before release, two central tail feathers were collected for stable isotope analysis. The blood was taken via brachial vein puncture using a 27-gauge needle and collected using a 70-μL capillary tube coated with anticoagulant (Winder and Emslie, 2012). After being transferred to labelled 1.5-mL Eppendorf tubes, cellular and plasma fractions were separated at 7000 rpm using a portable centrifuge (Scilogex® Model D1008 EZeeMini, Rocky Hill, USA) within 10 minutes of sampling. Blood plasma was transferred to a cryovial and stored on dry ice or in a liquid nitrogen CryoShipper until transferred to an ultra-cold freezer at −80°C. Each field site in 2018 was sampled for insects opportunistically to establish a baseline for stable isotope samples using a sweep net over vegetation at adjacent habitats next to the colonies. Adjacent habitats were open fields that Bank Swallow used as foraging habitat.

### Stable isotope analyses

Feather samples (2017–2018: *n* = 175 individuals) were soaked in 2:1 chloroform:methanol overnight, rinsed and dried in a fumehood at ambient temperature. Feather barbs from the distal end of the feather were analysed for stable hydrogen (δ^2^H) isotopes by weighing subsamples (~0.35 mg) into silver capsules (Mettler Toledo® XP6 Excellence Plus XP Micro Balance, Greifensee, CHE). Samples were assayed for stable hydrogen (δ^2^H) isotopes at the LSIS-AFAR stable isotope facility at the University of Western Ontario using a UNI-Prep (Eurovector, Milan, Italy) heated carousel (60°C) coupled with a Eurovector elemental analyser and combusted at 1350°C on a glassy carbon reactor. Resultant H_2_ gas was analysed on a coupled Thermo Delta V Plus (Thermo Scientific®, Bremen, DEU) continuous-flow isotope ratio mass spectrometer via a Conflo device (Thermo Scientific®). Sample results were expressed in the standard delta (δ) notation in parts per thousand (‰) deviation from in-house keratin standards (CBS: −197‰; KHS: −54.1‰). The δ^2^H value of the non-exchangeable H fraction was derived according to the comparative equilibration approach (Wassenaar and Hobson, 2003). Based on within-run (*n* = 5 each) keratin standards, measurement error was estimated to be ±2‰. All δ^2^H values were expressed relative to Vienna Standard Mean Ocean Water.

For δ^13^C and δ^15^N values, the same solvent-rinsed feather samples (2017–2018: *n* = 174 individuals) were weighed (~1.0 mg) into tin capsules and submitted to the Environment and Climate Change Canada Stable Isotope Laboratory in Saskatoon, Canada. Encapsulated feather was combusted at 1030°C in a Carlo Erba NA1500 or Eurovector 3000 elemental analyser. The resulting N_2_ and CO_2_ were separated chromatographically and introduced to an Elementar isoprime or an Nu Instruments Horizon isotope-ratio mass spectrometer. We used two reference materials to normalize the results to VPDB and AIR: BWBIII keratin (δ^13^C = −20.18, δ^15^N = +14.31‰, respectively) and PRCgel (δ^13^C = −13.64, δ^15^N = +5.07‰, respectively). Within run (*n* = 5 each) precisions as determined from both reference and sample duplicate analyses were ±0.1‰ for both δ^13^C and δ^15^N.

### Faecal DNA metabarcoding

For faecal matter DNA metabarcoding, we sent samples stored in 95% ethanol to the Canadian Center for DNA Barcoding at the University of Guelph (see Moran *et al.*, 2019, for the detailed protocol). There, faecal samples were homogenized and prey DNA was extracted and amplified using arthropod-specific primers. Each sample was tagged with Ion Xpress universal molecular identifiers and sequenced by an Ion Torrent PGM high-throughput sequencer. Sequence reads were associated to their source sample and low-quality reads were removed (QV20 minimum quality). Reads were adjusted by removing the primer and adapter sequences and filtering by a minimum of 100 base-pair lengths. Adjusted reads were compared with the Barcode of Life Database reference library and identified using the basic local alignment search tool. Identification was accepted if 50 reads or more matched the reference sequence (having 95% matches across 100 base-pairs minimum). Of 220 faecal samples, 171 were returned with prey identifications to a minimum taxonomic resolution of family.

### FA analyses

Plasma samples for FA analyses first underwent a solvent extraction (based on Bligh and Dyer, 1959; see Supplementary Material for the detailed protocol). Briefly, we mixed the blood plasma and a 17:0 internal standard with 2:1 chloroform:methanol containing butylated hydroxytoluene in a culture tube. Following centrifugation, the supernatant was mixed with 0.25% potassium chloride in new culture tubes and were placed in a 70°C water bath. Methanolic hydrogen chloride was added to the filtered organic phase and placed in a 90°C oven. Then, the top layer of an ultrapure water and hexane mixture was transferred to gas chromatograph (GC) vials. Dimethoxypropane was added to the hexane pool and dried under N_2_. Finally, hexane was added and collected into an insert that was placed back into the GC vials.

On a carousel, GC vials were loaded and samples were analysed by a GC/flame ionization detector (Agilent Technologies® 6890N G1530N, Santa Clara, USA) equipped with a DB23 column (Agilent DB23 122-2332, Santa Clara, USA; see Supplementary Material for temperature settings). For quality control, a blank of 100-μL dichloromethane and two standards (Supelco® PUFA and 37 components) were run at the beginning of a sampling week. The retention times of PUFA and 37 component standards were averaged to create a library of known FA peaks. The distinct peaks of each sample chromatograph were compared with the retention times in the library to identify known FAs. Relative to an internal standard 17:0, FA concentration and mass percent of each FA identified were calculated (2017–2018: *n* = 100).

### Statistical analyses

To evaluate if Bank Swallow nesting habitat affected diet composition and quality, we compared birds from inland pits to those from lakeshore colonies with respect to their feather stable isotope values, faecal prey composition and plasma FA profiles. All figures and statistical analyses were performed with RStudio Version 1.2.1335 and R 3.6.0 statistical software (RStudio Team, 2018; R Core Team, 2019). Significance level was established at *P* < 0.05.

To evaluate the effects of habitat type on stable isotope values and FA mass percentages, we used linear mixed-effect models (LMMs) fitted with the nlme package (Pinheiro *et al.*, 2019). Model selection of all LMMs used Akaike information criterion (AIC) corrected for small sample size from the R package MuMIn (Bartoń, 2019), and residuals were verified for normality, equal variance, leverage and collinearity. Julian date was collinear with site and was not included in any modelling. All LMMs had site as a random effect. To evaluate the effects of habitat type on prey items consumed and FA profiles, we used distance-based redundancy analyses (dbRDAs) fitted with the vegan package (Oksanen *et al.*, 2019). An automated model selection for the dbRDAs used AIC and *P*-values. Refer to Supplementary Material for model selection and model summaries.

For δ^2^H values, our LMM predictors included distance from Lake Erie, habitat type (inland versus lakeshore) and year. Using the package SIBER (Jackson *et al.*, 2011) for Bayesian statistics, we created ellipses of stable isotope values (δ^2^H, δ^13^C, δ^15^N) in juvenile feathers and their surface ellipse area were used to identify group clusters and the extent of overlap in ellipses between habitat and year. Another LMM compared stable isotope values (δ^2^H, δ^13^C, δ^15^N) of chironomids with those of terrestrial dipterans collected from our sweep net samples. For faecal samples, we converted prey items from the faecal metabarcoding for each sample into a presence/absence data matrix. We carried out a dbRDA for juvenile and adult faecal samples using Jaccard’s distance, which ignores the absent/absent possibilities, to identify differences in prey by habitat and year. Another dbRDA assessed the patterns in juvenile plasma FA profiles by habitat and year, using Euclidean distance for the total FA concentration and individual FA mass percentages. We also individually modelled EPA using an LMM for diet quality differences between habitat and year.

## Results

To evaluate dietary differences in Bank Swallows, we used juvenile tail feathers for stable isotopes, juvenile blood plasma for FAs and both adult and juvenile faecal matter for DNA metabarcoding. We captured 3771 Bank Swallows in 2017 and 2137 in 2018. We sampled tail feathers from 47 juveniles in 2017 and 128 juveniles in 2018. An additional 41 juveniles were sampled for the blood in 2017 and 59 juveniles in 2018. We opportunistically collected faecal matter and analysed those of 93 adults and 17 juveniles in 2017 and 50 adults and 7 juveniles in 2018.

### Stable isotopes

**Figure 2:**
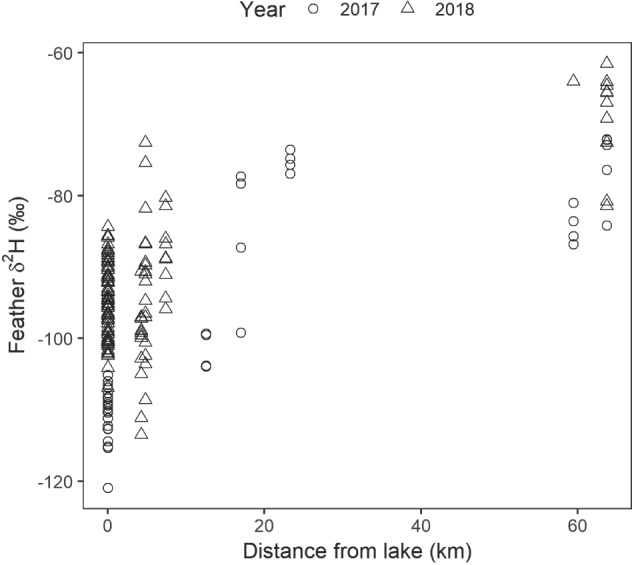
Relationship between feather δ^2^H_f_ (‰) and distance (km) from Lake Erie for juvenile Bank Swallows sampled in 2017 (*n* = 47) and 2018 (*n* = 128).

Juvenile feather δ^2^H_f_ values at aggregate pits differed from those at lakeshore sites. Distance of each colony from Lake Erie had a significant positive effect on δ^2^H_f_ for juveniles, in which every 1 km from the lake increased δ^2^H_f_ by 0.28‰ (LMM; t_14_ = 2.84, *P* = 0.01; Fig. 2). Overall, lakeshore juveniles had lower mean δ^2^H_f_ values than inland juveniles by 16‰ (LMM; t_14_ = 3.80, *P* < 0.01; Fig. 2). There was an interaction between habitat and year (LMM; t_156_ = −2.98, *P* < 0.01; Fig. 2), in which the δ^2^H_f_ difference between habitats was larger in 2017 than 2018.

**Figure 3:**
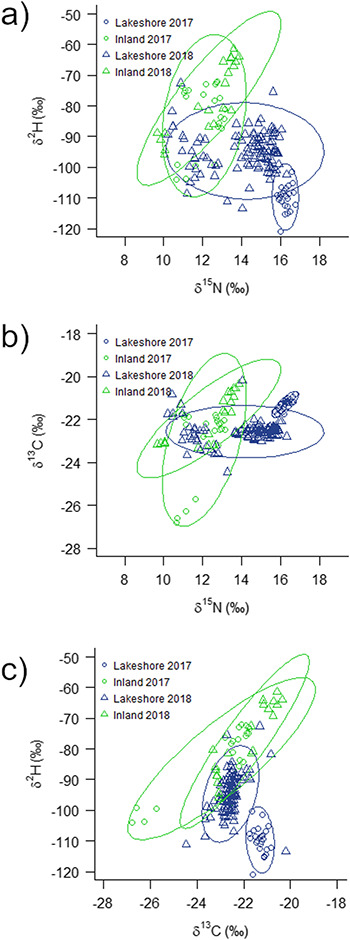
Biplots of juvenile Bank Swallow feather (a) δ^2^H_f_ versus δ^15^N_f_, (b) δ^13^C_f_ versus δ^15^N_f_ and (c) δ^2^H_f_ versus δ^13^C_f_ (‰) for lakeshore (*n* = 131) and inland (*n* = 43) sites in 2017 (*n* = 47) and 2018 (*n* = 127). Bayesian ellipses were grouped by habitat and year at 95% confidence interval.

Biplots comparing juvenile δ^2^H_f_, δ^13^C_f_ and δ^15^N_f_ values for 2017 and 2018 revealed that lakeshore birds consistently occupied different isotopic spaces from inland birds (Fig. 3). Bayesian ellipses showed that 2017 lakeshore juveniles were isotopically different and had no overlap with inland juveniles (Fig. 3). Area of ellipses corresponding to inland and lakeshore colonies in 2018 overlapped by only 12–24% for δ^2^H_f_ versus δ^15^N_f_ and for δ^13^C_f_ versus δ^15^N_f_ biplots (Fig. 3). Isotopic ellipses for inland breeders were larger and overlapped by 40–46% between years, showing more variability in isotopic values compared with the lakeshore birds that were more tightly clustered with little overlap (0–5%) between years (Fig. 3).

Isotopic values of potential insect prey in 2018 revealed that aquatic emergent chironomids (midges) differed fromterrestrial dipterans (flies) with lower mean δ^2^H_i_ (−165.3 ± 34.2‰, n_Chir_ = 6 versus -127.7 ± 16.9‰, n_T.Dipt_ = 9; LMM; t_13_ = 2.85, *P* = 0.01). Chironomids had higher mean δ^15^N_i_ (11.4 ± 2.5‰, n_Chir_ = 5 versus 5.5 ± 2‰, n_T.Dipt_ = 10; LMM; t_13_ = −4.90, *P* < 0.01) than terrestrial dipterans but did not significantly differ in δ^13^C_i_ (−26.5 ± 1.2‰, n_Chir_ = 5 versus -24.1 ± 2.8‰, n_T.Dipt_ = 10; LMM; t_13_ = 1.79, *P* = 0.10).

### Faecal DNA metabarcoding

Faecal DNA metabarcoding identified 12 orders and 77 families in adult and juvenile Bank Swallow faecal samples. The most common orders found included Diptera (flies; 68% of samples), Coleoptera (beetles; 36%), Hymenoptera (sawflies, wasps, bees and ants; 14%), Hemiptera (true bugs; 12%) and Lepidoptera (butterflies and moths; 9%). The remaining orders were each found in 3% or less of samples. Diptera had 30 identified families that were divided into two groups, aquatic/semi-aquatic dipterans and other terrestrial dipterans (Williams and Feltmate, 1992). The most commonly found families were Chironomidae (non-biting midges; 91% of samples), Anthomyiidae (root maggot flies; 13%), Dolichopodidae (long-legged flies; 12%), Sphaeroceridae (small dung flies; 9%), Limoniidae (type of crane fly; 8%), Chloropidae (fruit flies; 6%) and Drosophilidae (fruit flies; 6%). All other families were each present in 4% of samples or less. The orders Psocodea (lice), Sarcoptiformes and Trombidiformes (mites) were removed as they were found in few samples and likely not foraged insects. Samples with missing habitat or year information were omitted (*n* = 4).

Faecal samples were separated into two groups in ordination space, such that 2017 lakeshore birds differed from all other birds in terms of insect prey groups (orders and aquatic dipteran families; Supplementary Fig. S1). Chironomidae (Chir) were associated with the majority of 2017 lakeshore birds, while terrestrial dipterans (T.Dipt), Coleoptera (Cole), Anthomyiidae (Anth) and Hemiptera (Hemi) were associated with inland birds, but lakeshore birds in 2018 were more variable in terms of the prey they consumed. The metabarcoding ordination explained 8% of the variation in the presence of insect groups. The first axis significantly divided faecal samples, but the second axis had no clear pattern (dbRDA; axis1: *P* < 0.01, axis2: *P* = 0.06). As described above, there was a significant interaction between year and habitat (dbRDA; F_1_ = 2.57, *P* = 0.02), whereby diet composition differed more between lakeshore and inland birds in 2017 than 2018.

### FA profiles

**Figure 4:**
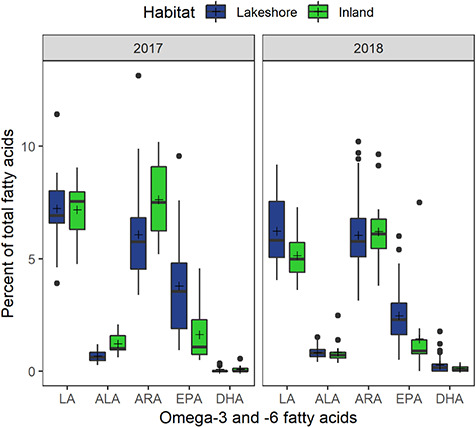
Boxplot of omega-3 and -6 FAs (%) in juvenile Bank Swallow blood plasma sampled in 2017 (*n* = 41) and 2018 (*n* = 59) between lakeshore (*n* = 65) and inland (*n* = 35) sites.

FA profiles of juvenile blood plasma were separated in ordination space by habitat and year, though a group of 2018 lakeshore juveniles overlapped with inland juveniles (see Supplementary Fig. S2). The majority of FAs were clustered in the middle of the ordination, indicating no association to group. Nonetheless, we identified some key FAs associated with nesting habitat (Supplementary Table S2). Lakeshore juveniles were associated with 18:1n7 vaccenic acid and 20:5n3 EPA, while inland juveniles were associated with 18:1n9 oleic acid and 20:4n6 arachidonic acid (ARA). The FA ordination explained 16% of the total variation in FA percentages, where the first axis significantly divided birds by year and the second axis significantly divided birds by habitat (dbRDA; axis1: *P* < 0.01, axis2: *P* = 0.01; Fig. 4). Specifically, lakeshore birds differed significantly from inland birds in FA profiles (dbRDA; F_1_ = 4.08, *P* < 0.01). Controlling for habitat type, juveniles of each year were significantly different from each other in terms of FA profiles (dbRDA; F_1_ = 14.74, *P* < 0.01).

We assessed diet quality by evaluating mean percentages of omega-3 and -6 FAs. Juvenile plasma at lakeshore sites was twice as high in EPA than inland birds but variable in plasma LA, ALA and ARA depending on the year (Fig. 4). Lakeshore juveniles had significantly higher blood plasma EPA than inland juveniles (t_14_ = −2.81, *P* = 0.01; Fig. 4). We also found that blood plasma EPA was higher in 2017 than in 2018 when controlled for habitat type (t_14_ = −2.66, *P* < 0.01; Fig. 4).

## Discussion

Using a variety of endogenous molecular markers, our study clearly separated the diet of Bank Swallows nesting at inland aggregate mining pits from those nesting at lakeshore sites. In particular, we found that lakeshore birds consumed more aquatic emergent insects compared with birds inhabiting inland areas as indicated by stable isotope analysis of feathers and faecal DNA metabarcoding. Aquatic emergent insects are vectors of both nutrients (e.g. EPA and DHA) (Hixson *et al.*, 2015; Twining *et al.*, 2018a) and contaminants (e.g. polychlorinated biphenyls and methylmercury) (Echols *et al.*, 2004; Walters *et al.*, 2008; Jackson *et al.*, 2015) from aquatic systems to terrestrial consumers. Dietary differences were also manifested in plasma PUFA profiles of Bank Swallows, such that lakeshore birds consuming chironomids clearly had higher levels of omega-3 EPA. In addition, our three markers reveal yearly differences such that 2017 birds had a more aquatic-based diet than 2018 birds. Yearly, differences were particularly pronounced in lakeshore birds suggesting that they may profit from years of productive chironomid emergences.

Establishing the presence of an ecological trap requires a clear demonstration that such sites are preferred and act as population sink (Battin, 2004). Aggregate pits in this sense may not be ecological traps per se (Burke *et al.*, 2019). However, we have demonstrated that there is likely a nutritional cost related to diets provisioned to nestlings at inland versus lakeshore sites. In this sense, we prefer the term nutritional trap. The quantity of available aerial insects fuels nestling growth, but there is evidence that diet quality is more important than quantity in supporting healthier individuals (Quinney *et al.*, 1986; Twining *et al.*, 2016b). In New Brunswick, three swallow species did not increase nestling survival or mass with increasing insect abundance (Imlay *et al.*, 2017). Twining *et al*. (2016b) found that tree swallow nestling mass growth rate increased more if food quality was elevated instead of quantity. A recent study on Bank Swallows in our study region reported that aggregate pits and lakeshore colonies had no differences in breeding success and birds in pits had fewer ectoparasites (Burke *et al.*, 2019). However, we have demonstrated a potential nutritional disadvantage for birds nesting at inland pits and provide evidence that aggregate pits may be acting as ‘nutritional traps’. Diet quality clearly fluctuates with landscape and our approach strongly suggests that it is possible to combine dietary markers to create ‘nutritional landscapes’ relevant to conservation.

### Stable isotopes

Previous research has used tissue δ^13^C values in consumers to delineate their dependence on aquatic versus upland food webs (Doucett *et al.*, 1996; Clementz and Koch, 2001; Walters *et al.*, 2008; also see Macdade *et al.*, 2011). Within plants of similar photosynthetic pathway, terrestrial organic matter δ^13^C is relatively uniform but aquatic primary producer δ^13^C can be highly variable depending on site and local conditions (Doucett *et al.*, 1996; Clementz and Koch, 2001; Finlay, 2004). We found that feather δ^2^H values showed a larger isotopic range separating aquatic and inland insect prey compared with δ^13^C values and are likely to be more universally appropriate for assessing aquatic versus terrestrial diets (Voigt *et al.*, 2015; Vander Zanden *et al.*, 2016). Aquatic organic matter δ^2^H values tend to be consistently lower than terrestrial organic matter (Doucett *et al.*, 2007). Similarly, chironomid larvae burrowing into the sediment may consume biomass derived from methanogenesis, which has distinctly low δ^13^C and δ^2^H values (Grey, 2016). We also found a gradient in Bank Swallow δ^2^H_f_ values that increased steadily as nesting site became more distant from Lake Erie. Swallows nesting in pits closer to the lake had low δ^2^H_f_ similar to those found at natural lakeshore sites and so were likely accessing aquatic emergent insects that were dispersed by onshore winds (see MacKenzie and Kaster, 2004).

Although δ^2^H_f_ analyses proved to be a powerful indicator of aquatic emergent insect use by swallows, our multi-isotope approach was also useful. Bank Swallows raised in lakeshore and inland habitats were separated in isotope space as indicated by the δ^2^H_f_, δ^15^N_f_ and δ^13^C_f_ biplots. The analyses further indicated that aquatically derived diets were typically associated with higher δ^15^N_f_ values. Periphyton in aquatic systems can have higher δ^15^N values than primary producers in terrestrial systems (Jardine *et al.*, 2012). Another possibility is that Bank Swallows foraging on aquatic emergent insects reflect elevated δ^15^N values of fertilizer-based nitrogen concentrated in aquatic systems. Sediment cores from Lake Erie show a gradual lake eutrophication and increasing δ^15^N from anthropogenic nutrient inputs (Lu *et al.*, 2010). The deposition of excess fertilizers and draining of fields into aquatic systems can elevate δ^15^N of larger waterbodies and surrounding consumers (Harrington *et al.*, 1998; Hebert and Wassenaar, 2001; Bateman *et al.*, 2005).

### Faecal DNA metabarcoding

Faecal DNA analyses were useful in identifying the diversity of insects used by Bank Swallows. Diet was predominantly composed of aerial insects from the Diptera and Coleoptera orders. As expected, this technique indicated that Bank Swallows nesting at lakeshore sites foraged more on chironomids, especially in 2017. By contrast, inland birds fed more on terrestrial dipterans, coleopterans, root-maggot flies (Anthomyiidae) and hemipterans. Comparative data are scarce, but Bank Swallows in the river banks and quarries of Scotland foraged similarly on the orders Diptera (69%), Hemiptera (13%), Coleoptera (11%) and Hymenoptera (5%) (Waugh, 1979). Overall, our results confirm that Bank Swallows forage on taxonomically diverse ‘aerial plankton’. Proximity to the lakeshore provides swallows the opportunity to profit from productive aquatic insect emergences. The availability of emergent insects inland, however, is likely dependent on wind conditions and direction during any given period.

### FA profiles

FA profiles of juvenile Bank Swallows showed differences between those raised at lakeshore and inland sites as well as between years. Lakeshore juveniles had more 18:1n7 vaccenic acid and 20:5n3 EPA in blood plasma, while inland birds had more 18:1n9 oleic acid and 20:4n6 ARA in blood plasma. We found strong support that lakeshore juvenile birds acquired a higher-quality diet compared with inland birds, as indicated by plasma omega-3 EPA. The percent composition of FAs depends on tissue (Twining *et al.*, 2016b, 2018a). The relative percent of plasma EPA in swallow chicks was double at lakeshore sites compared with inland sites. Our results are comparable with those of great tits (*Parus major*) that had switched winter feeder diets to summer insect diets (Andersson *et al.*, 2015). Plasma EPA we found for lakeshore Bank Swallows was generally higher than values reported for other passerines (Isaksson *et al.*, 2017). By feeding on aquatic emergent insects, terrestrial consumers can acquire higher amounts of PUFAs (Hixson *et al.*, 2015; Twining *et al.*, 2018a). Twining *et al.* (2016b, 2018a) found that higher dietary EPA increases nestling health and fledging success. Despite the higher plasma EPA at lakeshore sites, Bank Swallows had low plasma DHA in both habitat types. It is uncertain if this means that Bank Swallows were unable to acquire DHA or that incorporated DHA was simply not present in the blood. Borisova *et al.* (2016) found that adult chironomids had more EPA than larvae but DHA was low at both life stages. Chironomids are a large source of prey biomass and PUFAs such as ALA, LA and EPA but they provide little DHA compared with other species [e.g. phantom midges (*Chaoborus flavicans*) Makhutova *et al.*, 2017; Martin-Creuzburg *et al.*, 2017]. Alternatively, DHA is more abundant in brain tissues than other tissues and may simply not be reflected in Bank Swallow blood plasma (Twining*et al.*, 2018a).

Understanding the potential benefits of omega-3 FAs to songbirds is a relatively new area of research that requires further investigation. In addition to promoting nestling growth and fledging success, avian studies have yet to explore the advantages of high EPA and DHA as reported in humans (e.g. supporting the brain and retinal development, providing anti-inflammatory properties and preventing disease; Simopoulos, 2011). Great tits in Sweden had higher omega-3 FAs in winter in rural habitats than urban habitats, while urban habitats had more ARA (Andersson *et al.*, 2015). Andersson *et al.* (2015) suggested that the negative effects of high pollutant exposure in urban areas are potentially exacerbated by elevated pro-inflammatory responses from high ARA, while the high omega-3 FAs aided in anti-inflammatory responses.

Bank Swallows at our inland sites had higher plasma oleic acid and lower plasma vaccenic acid than lakeshore birds. In Pakistan, lake-dwelling cormorants (*Phalacrocorax niger*) had high concentrations of vaccenic acid in eggs (Ramírez*et al.*, 2009). Vaccenic acid is typically found in bacteria, and so lake sediments supporting anaerobic bacteria could elevate these FA concentrations in aquatic systems (Ramírez *et al.*, 2009). Chironomids in Siberian lakes contain high levels of oleic and vaccenic acids but, as selective feeders, chironomid FA profiles are dependent on species-specific preferences for bacterial food or other food items such as algae or diatoms (Makhutova *et al.*, 2017). Oleic acid levels can vary greatly across species but have been found to be high in several terrestrial insects (Womeni *et al.*, 2009; Rumpold and Schlüter, 2013; Pino Moreno and Ganguly, 2016). Overall, insects can be high in oleic acid, whereas vaccenic acid is typically found in trace amounts (Rumpold and Schlüter, 2013).

### Summary

A recent analysis of mayfly emergence flights using radar in the western Lake Erie basin and waterways estimated 3000 tons of mayfly biomass from a single emergence event (Stepanian *et al.*, 2020). However, despite producing billions of mayflies, recent mayfly productivity from 2012 to 2019 had declined by more than 50% (Stepanian *et al.*, 2020). Likewise, chironomids have steeply declined since 1981 (Soster*et al.*, 2011). These decreases in insect biomass can drive proportional declines in aerial insectivore populations (Møller, 2019). We advocate formal consideration of nutrition in the conservation and management of species. While it is generally recognized that food availability is an important component of suitable habitat, understanding prey quality and distribution formally in terms of ‘nutritional landscapes’ could aid in animal conservation (Twining *et al.*, 2016b, 2018a; Rubin*et al.*, 2019; Spiller and Dettmers, 2019). Our study shows the importance of conserving high-quality lakeshore habitats and supporting productive aquatic insect emergences. Likewise, management of aquatic systems should include the mitigation of pollutants that can negatively affect the aquatic fauna and toxic compounds that can similarly transfer from aquatic to terrestrial systems. The preservation of aquatic systems will support not only riparian specialists such as Bank Swallows but also other insectivorous songbirds using the same riparian habitat (Blancher and McNicol, 1991; Brown *et al.*, 2002; Deschênes *et al.*, 2003; Twining *et al.*, 2018a; Jackson*et al.*, 2020) and similar forested riparian habitats (Gray, 1993; Rottenborn, 1999; Lussier *et al.*, 2006; Pennington*et al.*, 2008; Rubin *et al.*, 2019). Understanding foraging ranges of Bank Swallows from inland and lakeshore sites using radiotelemetry (see Evans *et al.*, 2020) will also help evaluate costs and benefits of using natural versus aggregate pits in this species.

Our study underlines the power of using a diverse suite of analytical methods to examine avian diets. Stable isotope approaches will benefit from greater emphasis on isotopic values of the prey base and how these are influenced by landscape practices (Peterson *et al.*, 1993; del Rosario *et al.*, 2002; Deines *et al.*, 2009). FA analyses are a powerful means of tracing the relative importance of aquatic-emergent versus terrestrial insect biomass to nestlings and adults, and future studies should better evaluate residence times of these FAs in various tissue components (Foglia *et al.*, 1994; Wang *et al.*, 2010), especially in blood plasma versus blood cells. Our use of plasma FA profiles presumably reflected short-term diet and longer periods of dietary assimilation are possible using assays of the cellular blood component.

## Supplementary Material

SUPPLEMENTARY_REVISED_clean_copy_coaa140
