# Supplementary material for: Nutritional consequences of breeding away from riparian habitats in Bank Swallows: new evidence from multiple endogenous markers
Source: Conserv Physiol. 2021 Jan 19;9(1):coaa140. doi: 10.1093/conphys/coaa140 (PMC7836397; doi:10.1093/conphys/coaa140)
Supplement: SUPPLEMENTARY_REVISED_clean_copy_coaa140 [file supplementary_revised_clean_copy_coaa140.doc]

# **Supplementary material**

**Table S1:** Coordinates of field sites, their distance from Lake Erie, and the year sampled.

| ID | Site | Coordinates (Lat, Lon) | Distance (km) | Habitat | Year |
| --- | --- | --- | --- | --- | --- |
| Bd | Blythedale | 43.237198, -80.948718 | 63.69 | Pit | 2017-2018 |
| Em | Embro | 43.194902, -80.940330 | 59.43 | Pit | 2017-2018 |
| Co | 2^nd^Concession | 42.818052, -80.546288 | 23.30 | Pit | 2017 |
| Wf | Waterford | 42.916073, -80.320121 | 16.96 | Pit | 2017 |
| Si | Simcoe | 42.855651, -80.341145 | 12.51 | Pit | 2017 |
| PV | Pleasant Valley | 42.726890, -81.063970 | 7.36 | Pit | 2018 |
| Cu | Cultus | 42.623162, -80.599831 | 4.79 | Lake | 2017-2018 |
| Li | Lipsit | 42.804677, -80.267088 | 4.24 | Lake | 2017-2018 |
| Al | Allenson | 42.639689, -80.773568 | 0 | Lake | 2018 |
| Bl | Bolin | 42.622835, -80.723627 | 0 | Lake | 2017-2018 |
| Bs | Bossuyt | 42.661656, -80.981239 | 0 | Lake | 2017-2018 |
| Cr | Crosby | 42.661787, -81.077609 | 0 | Lake | 2017-2018 |
| Go | Godby | 42.629546, -80.733805 | 0 | Lake | 2018 |
| Gu | Gunn | 42.600942, -80.672197 | 0 | Lake | 2017-2018 |
| Ha | Hayhoe | 42.660727, -80.906784 | 0 | Lake | 2017-2018 |
| Pa | Passmore | 42.661382, -80.914303 | 0 | Lake | 2017 |
| Wl | Wall | 42.611888, -80.697900 | 0 | Lake | 2017 |

**Table S2:** Mean total fatty acid (mmol/L) and fatty acid percentage of juvenile Bank Swallows.

|  | Mean (± SD) | | | | |
| --- | --- | --- | --- | --- | --- |
|  | Lakeshore | | Inland | | |
| Fatty acid | 2017 | 2018 | | 2017 | 2018 |
| Total FA | 20.24 (4.00) | 26.52 (5.80) | | 20.00 (4.16) | 27.77 (4.06) |
| 14:0 | 1.25 (0.68) | 2.69 (2.69) | | 0.99 (0.63) | 2.89 (2.08) |
| 15:0 | 0.49 (0.28) | 0.32 (0.45) | | 0.22 (0.19) | 0.35 (0.50) |
| 16:0 | 35.71 (6.33) | 36.99 (4.51) | | 24.36 (5.13) | 38.22 (4.01) |
| 16:1n7 | 4.12 (2.20) | 2.15 (0.71) | | 2.80 (1.01) | 2.96 (1.50) |
| 17:1 | 0.28 (0.18) | 0.09 (0.20) | | 0.10 (0.14) | NA |
| 18:0 | 25.74 (4.87) | 28.49 (4.96) | | 26.37 (4.08) | 27.45 (4.75) |
| 18:1n9 | 7.38 (3.10) | 8.81 (4.29) | | 12.61 (3.44) | 10.86 (2.25) |
| 18:1n7 | 3.98 (1.43) | 2.36 (0.80) | | 1.95 (1.23) | 1.20 (0.27) |
| 18:2n6 | 7.23 (1.59) | 6.22 (1.44) | | 7.17 (1.18) | 5.14 (1.08) |
| 18:3n6 | 0.59 (0.28) | 0.31 (0.11) | | 0.56 (0.29) | 0.32 (0.08) |
| 18:3n4 | 0.03 (0.06) | <0.01 (0.02) | | 0.09 (0.15) | 0.06 (0.11) |
| 18:3n3 | 0.66 (0.27) | 0.81 (0.25) | | 1.21 (0.41) | 0.84 (0.51) |
| 18:4n3 | 0.09 (0.14) | NA | | 0.04 (0.08) | NA |
| 20:0 | 0.28 (0.10) | 0.31 (0.09) | | 0.24 (0.12) | 0.29 (0.10) |
| 20:3n6/21:0 | 0.29 (0.15) | 0.12 (0.15) | | 0.35 (0.14) | 0.27 (0.11) |
| 20:4n6 | 6.07 (2.23) | 6.04 (1.72) | | 7.62 (1.55) | 6.21 (1.63) |
| 20:5n3 | 3.78 (2.21) | 2.46 (1.24) | | 1.62 (1.20) | 1.43 (1.74) |
| 22:5n3 | 0.64 (0.20) | 0.54 (0.19) | | 0.44 (0.26) | 0.35 (0.14) |
| 24:0 | 1.31 (0.46) | 1.13 (0.45) | | 1.24 (0.36) | 1.16 (0.35) |
| 22:6n3 | 0.08 (0.14) | 0.27 (0.37) | | 0.07 (0.14) | 0.12 (0.14) |

**Table S3:** Summary of model selection using the MuMIn package, showing the top 3 models. All LMMs have site as a random effect.

| Model | AIC | AICc | Weight |
| --- | --- | --- | --- |
| δ^2^H ~ Distance + Year * Habitat | 1133 | 1134 | 0.699 |
| δ^2^H ~ Year * Habitat | 1135 | 1136 | 0.295 |
| δ^2^H ~ Distance + Year + Habitat | 1144 | 1145 | 0.004 |
| Juvenile EPA ~ Year * Habitat | 352 | 353 | 0.551 |
| Juvenile EPA ~ Year + Habitat | 354 | 354 | 0.243 |
| Juvenile EPA ~ Habitat | 355 | 356 | 0.124 |

**Table S4:** Summary of model selection using p-values and AIC for the dbRDAs. No collinearity was identified. Automated backward selection drops non-significant terms.

|  | AIC | | |  | p-value | | |
| --- | --- | --- | --- | --- | --- | --- | --- |
| Term | Drop 1 | Drop 2 | check |  | Drop 1 | Drop 2 | check |
| Fecal DNA |  |  |  |  |  |  |  |
| Yr*Hbt | 694.24 |  | 694.24 |  | 0.03 |  | 0.02 |
| Juvenile FA |  |  |  |  |  |  |  |
| Yr*Hbt | 464.41 |  |  |  | 0.17 |  |  |
| Habitat |  | 466.53 | 466.53 |  |  | 0.02 | < 0.01 |
| Year |  | 476.55 | 476.55 |  |  | < 0.01 | < 0.01 |

**Table S5:** Summary of the final LMM for δ^2^H_f_ of juvenile Bank Swallow tail feathers with site as a random effect. Also shown are the approximate 95% confidence intervals, the model R-squared and adjusted R-squared.

| Term | N | Lower | Upper | SE | t | p-value | R^2^ | adjR^2^ |
| --- | --- | --- | --- | --- | --- | --- | --- | --- |
| Intercept | 175 | -114.99 | -106.96 | 2.03 | -54.60 | < 0.01 | 0.73 | 0.73 |
| Distance |  | 0.07 | 0.49 | 0.10 | 2.84 | 0.01 |  |  |
| Year 2018 |  | 12.94 | 19.48 | 1.65 | 9.80 | < 0.01 |  |  |
| Habitat Inland |  | 7.16 | 25.75 | 4.33 | 3.80 | < 0.01 |  |  |
| 2018*Inland |  | -14.39 | -2.93 | 2.90 | -2.98 | < 0.01 |  |  |
| *Random ~Site |  |  |  | 5.03 |  |  |  |  |
| *Residuals |  |  |  | 5.57 |  |  |  |  |

*These terms are expressed with standard deviation

**Table S6:** Summary of the LMM for δ^2^H_f_, δ^15^N_f_, and δ^13^C_f_ of chironomids versus terrestrial dipterans with site as a random effect. Also shown are the approximate 95% confidence intervals, the model R-squared and adjusted R-squared.

| Term | N | Lower | Upper | SE | t | p-value | R^2^ | adjR^2^ |
| --- | --- | --- | --- | --- | --- | --- | --- | --- |
| δ^2^H_f_ |  |  |  |  |  |  |  |  |
| Intercept | 15 | -187.38 | -143.24 | 10.21 | -16.18 | < 0.01 | 0.38 | 0.38 |
| Insect group |  | 9.10 | 66.08 | 13.19 |  | 0.01 |  |  |
| *Random ~Site |  |  |  | 23.43 |  |  |  |  |
| *Residuals |  |  |  | 8.79 |  |  |  |  |
|  |  |  |  |  |  |  |  |  |
| δ^15^N_f_ |  |  |  |  |  |  |  |  |
| Intercept | 15 | 9.30 | 13.53 | 0.98 | 11.66 | < 0.01 | 0.65 | 0.65 |
| Insect group |  | -8.46 | -3.29 | 1.20 | -4.90 | < 0.01 |  |  |
| *Random ~Site |  |  |  | 2.05 |  |  |  |  |
| *Residuals |  |  |  | 0.77 |  |  |  |  |
|  |  |  |  |  |  |  |  |  |
| δ^13^C_f_ |  |  |  |  |  |  |  |  |
| Intercept | 15 | -28.79 | -24.16 | 1.07 | -24.70 | < 0.01 | 0.20 | 0.20 |
| Insect group |  | -0.49 | 5.19 | 1.31 | 1.79 | 0.10 |  |  |
| *Random ~Site |  |  |  | 2.24 |  |  |  |  |
| *Residuals |  |  |  | 0.84 |  |  |  |  |

*These terms are expressed with standard deviation

**Table S7:** Summary statistics for the dbRDA of prey items found in Bank Swallow fecal samples.

| Term | N | F stat | p-value | Proportion explained | Axis 1 p-value | Axis 2 p-value |
| --- | --- | --- | --- | --- | --- | --- |
|  | 167 |  |  | 0.08 | < 0.01 | 0.06 |
| Hbt*Yr |  | 2.57 | 0.02 |  |  |  |

**Table S8:** Summary statistics for the dbRDA of total fatty acid (mmol/L) and 21 identifiable fatty acids (%) in juvenile Bank Swallow blood plasma.

| Term | N | F stat | p-value | Proportion explained | Axis 1 p-value | Axis 2 p-value |
| --- | --- | --- | --- | --- | --- | --- |
|  | 100 |  |  | 0.16 | < 0.01 | 0.01 |
| Habitat |  | 4.08 | < 0.01 |  |  |  |
| Year |  | 14.74 | < 0.01 |  |  |  |

**Table S9:** Final LMM statistics for EPA of juvenile Bank Swallow blood plasma with site as the random effect. The 95% confidence intervals, model R-squared and adjusted R-squared are listed.

| Term | N | Lower | Upper | SE | t | p-value | R^2^ | adjR^2^ |
| --- | --- | --- | --- | --- | --- | --- | --- | --- |
| Intercept | 100 | 2.73 | 4.57 | 0.46 | 7.88 | < 0.01 | 0.41 | 0.42 |
| Year 2018 |  | -1.89 | -0.27 | 0.41 | -2.66 | < 0.01 |  |  |
| Habitat Inland |  | -3.50 | -0.47 | 0.71 | -2.81 | 0.01 |  |  |
| 2018*Inland |  | -0.18 | 2.43 | 0.66 | 1.71 | 0.09 |  |  |
| *Random ~Site |  |  |  | 1.10 |  |  |  |  |
| *Residuals |  |  |  | 1.18 |  |  |  |  |

*These terms are expressed with standard deviation


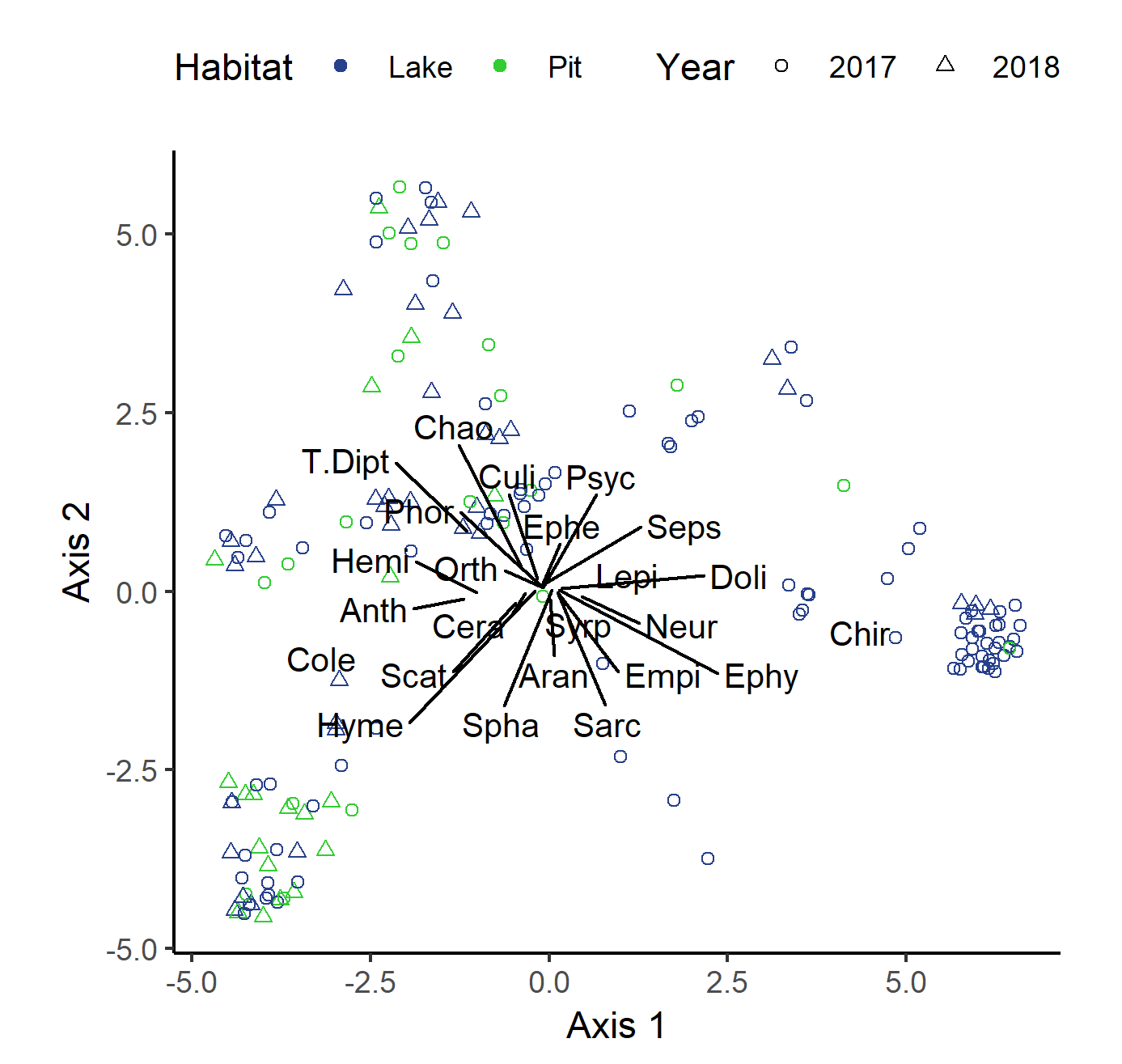


Figure S1: Prey items in fecal samples of Bank Swallows in a dbRDA. The ordination includes every lakeshore (n=127) and inland (n=40) bird sampled in 2017 (n=110) and 2018 (n=57). Insect labels and points were manipulated to avoid over-plotting and so, lines indicate the true position of a label.


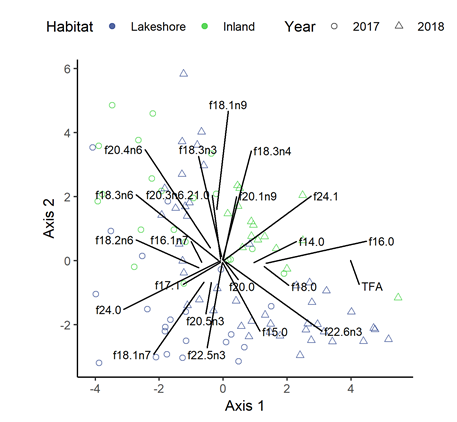


**Figure S2:** Total fatty acid (mmol/L) and individual fatty acids (%) of juvenile Bank Swallow blood plasma in a dbRDA. Juveniles were sampled in 2017 (n=41) and 2018 (n=59) among lakeshore (n=65) and inland (n=35) sites. Lines indicate the true position of a label.

**Fatty acid extraction protocol:**

In a culture tube, 10 μl of blood plasma, 20 μl of 17:0 internal standard (3 mg/mL), and 2 mL of 2:1 chloroform:methanol containing 0.01% butylated hydroxytoluene were mixed. Tubes were centrifuged at 3000 rpm for 10 minutes and the supernatant was transferred to clean tubes. After adding 1 mL of 0.25% potassium chloride, tubes were vortexed and placed in a 70 ºC water bath for 5 minutes. The bottom organic phase was filtered through glass wool into 2 mL glass vials and dried under N_2_ gas. To the vials, 200 μl of 0.5 M methanolic hydrogen chloride was added and swirled. Vials were placed in a 90 ºC oven for 30 minutes to obtain fatty acid methyl esters. Following the addition of 800 μl of ultrapure water and 500 μl hexane and shaken, the top hexane layer was transferred to GC vials. Hexane was added to the remaining water and transferred twice more. To the hexane pool, 100 μl of water scavenger dimethoxypropane was added and dried under N_2_. Finally, 100 μl of hexane was added, swirled, and transferred into an insert that collects the mixture into a smaller space. The insert was placed back into the GC vials and capped.

**Gas chromatograph/ flame ionization detector settings:**

GC vials were loaded on a carousel for fatty acid analysis by an Agilent 6890 gas chromatograph/ flame ionization detector. The injector needle was cleaned in dichloromethane, the injector temperature was set at 250 ºC, the flame ionization detector temperature was at 280 ºC, and the carrier gas flow rate of helium was set at 1.9 mL/min. The elution temperature began at 80 ºC for 2 minutes, increased up to 180 ºC over a 5 ºC min^-1^ rate, and held at 180 ºC for 5 minutes. Then, temperature was increased to 200 ºC over a 1 ºC min^-1^ rate with no holding temperature, followed by an increase to 240 ºC over 10 ºC min^-1^ rate, and a final holding at 240 ºC for 3 minutes (total of 54 minutes).
